# Colorimetric Sensor Array for White Wine Tasting

**DOI:** 10.3390/s150818197

**Published:** 2015-07-24

**Authors:** Soo Chung, Tu San Park, Soo Hyun Park, Joon Yong Kim, Seongmin Park, Daesik Son, Young Min Bae, Seong In Cho

**Affiliations:** 1 Department of Biosystems & Biomaterials Science and Engineering, Seoul National University, 1 Gwanak-ro, Gwanak-gu, Seoul 151-921, Korea; E-Mails: goodhahagigo@naver.com (S.C.); ecoloves@gmail.com (S.H.P.); tombraid@snu.ac.kr (J.Y.K.); marvelous@live.co.kr (S.P.); ftiu7957@snu.ac.kr (D.S.); 2 Department of Agricultural and Biosystems Engineering, University of Arizona, 1177 E. 4th St., Tucson, AZ 85721, USA; E-Mail: tusan.park@gmail.com; 3 Research Institute for Agriculture and Life Sciences, Seoul National University, 1 Gwanak-ro, Gwanak-gu, Seoul 151-921, Korea; 4 Korea Electrotechnology Research Institute, Ansan-si, Gyeonggi-do 426-910, Korea

**Keywords:** taste sensor, colorimetric, principle component analysis, artificial neural network

## Abstract

A colorimetric sensor array was developed to characterize and quantify the taste of white wines. A charge-coupled device (CCD) camera captured images of the sensor array from 23 different white wine samples, and the change in the R, G, B color components from the control were analyzed by principal component analysis. Additionally, high performance liquid chromatography (HPLC) was used to analyze the chemical components of each wine sample responsible for its taste. A two-dimensional score plot was created with 23 data points. It revealed clusters created from the same type of grape, and trends of sweetness, sourness, and astringency were mapped. An artificial neural network model was developed to predict the degree of sweetness, sourness, and astringency of the white wines. The coefficients of determination (*R^2^*) for the HPLC results and the sweetness, sourness, and astringency were 0.96, 0.95, and 0.83, respectively. This research could provide a simple and low-cost but sensitive taste prediction system, and, by helping consumer selection, will be able to have a positive effect on the wine industry.

## 1. Introduction

Taste sensor, a system composed of a sensor array and a data-processing unit, can be used to quantify and classify liquid media containing multiple taste components. The sensor array is a bundle of sensing elements that are non-selectively responsive to taste materials, and has been implemented with several transduction mechanisms uch as potentiometry [1,2,3], voltammetry [4,5], and conductivity [6,7]. Additionally, those were prevalently applied to discriminate beverages, and quantify sourness and saltiness [5,8,9,10,11]. In recent studies, the sensor arrays have been implemented with colorimetry that utilize the chemical dyes that are optically sensitive to taste materials. The taste sensor based on the colorimetry has advantages over the potentiometry as follows: (1) low cost; (2) sensitivity; and (3) a variety of the chemical dyes for each sensing element [12,13,14]. In addition, the contaminations of the membrane in the potentiometry can be eliminated in the colorimetry-based sensors. Different types of beers [12], soft drinks [13], rice wines [15], water [16], sweeteners [17], and red wines [18] were tested with a colorimetric sensor array, and their tastes were characterized and visualized on a taste map.

Wine is one of the most popular alcoholic beverages in the world. The amount of wine consumed in 2013 was reported as 23,367.4 million liters, which is a 1.4% increase from 2009. The global wine market forecasts that the market size will grow by 4.4% to $343.4 billion USD (United State Dollar) from 2013 to 2018 [19]. The amount consumed and market growth indicate the popularity of this market. Generally, wines are chosen because of their complexity, taste, and flavor. Classification of wines is very important because it is related to the quality of wines, adulteration, and control of the winemaking process, which connect directly to its economic value [20]. Additionally, detecting adulterations and authenticity of wine is a major issue in the industry [21]. However, characterization and discrimination is not an easy task due to the nature of wine taste, so these tasks fall to professional sommeliers. Unfortunately, variability between taste panelists can sometimes be more than 50% in terms of flavor and taste. Many attempts have been made to characterize wines analytically by taste and aroma using high performance liquid chromatography (HPLC) or gas chromatography-mass spectrometry (GC-MS) [22,23]. However, the cost of the laboratory, instruments, and skilled personnel is high, and their results are limited to the quantification of major elements and do not provide ‘taste’ results. Therefore, developing an objective, cheap, and reliable taste sensor, not only for tasting wine but for various food materials, is an attractive topic in sensor research [10,24,25,26,27,28].

In this study, we report the development of a colorimetric sensor array for white wine testing. Each sensing element of the sensor array is a polystyrene microbead conjugated with a chemical dye, which changes color with the material solution surrounding the microbead. Dyes were selected that are used frequently and are commercially available. The use of dye-conjugated particles prevents color change from being diluted or diffused by a loaded sample. The sensor array was fabricated by separately packing eight types of microbead in the open chambers of a single solid substrate to acquire the color of each microbead in a single shot of the still camera. The colorimetry results of the sensor array were applied to the classification of white wines, and the quantification of sweetness, sourness, and astringency in the wines was determined using principal component analysis (PCA) and an artificial neural network (ANN).

## 2. Experimental Section

### 2.1. White Wine Samples

Two different white wine sample sets were prepared for each PCA and ANN. The first set was from 23 original bottles purchased at a retail store (Table 1 and Supplement 1 for more details). The second set consisted of the 23 original samples and 22 mixtures of the original samples to prepare three different sample sets in order to avoid an over-fitting result from the ANN. None of the samples was from the same winery. All samples were tested on the colorimetric sensor array without any pre-treatment. The data from the first and second sample set were used for PCA and ANN, respectively.

**Table 1:** White wine sample list with grape species, vintage, and country of origin.

Grape Species	# of Bottles	Vintage	Country	Label
Chardonnay	8	2006, 2007, 2010, 2011, 2012	Chile, France, USA	Ch1-Ch8
Riesling	5	2010, 2011	German, New Zealand	Rl1-Rl5
Sauvignon blanc	4	2011, 2012	New Zealand	SB1-SB4
Pinot Grigio	1	2011	Italia	PG
Rivaner	1	2008	German	Rn
Torrontes	1	2007	Argentina	Tr
Moscato bianco	1	2011	Italia	MB
80% Semillion +20% Sauvignon blanc	1	2006	France	SS
70% Chardonnay +15% Pinot Grigio +15% Pinot Blanc	1	2011	Chile	CPP

### 2.2. Analysis of the Sweetness, Sourness, and Astringency of Wine Samples

High performance liquid chromatography (HPLC) was used to analyze the taste-related chemicals related to sweetness and sourness in white wine samples. Sucrose, glucose, and fructose were selected for identification of the sweetness content, and citric acid, malic acid, tartaric acid, succinic acid, lactic acid, and acetic acid were selected for identification of the sourness content. Each wine sample was filtered through 0.45 μm pore size syringe filter (Whatman, Buckinghamshire, UK) and tested by HPLC (HP1100, Agilent, CA, USA). Two different columns, Aminex 87-C, and Aminex 87-H (BioRad, CA, USA), were used to analyze the carbohydrate and organic acid content using 0.01 mol of NH_2_SO_4_ at 40 °C and deionized water at 80 °C for the mobile phase, respectively. The flow rate and injection volume for both columns were 0.5 mL/min and 5 μL.

The sweetness of each wine sample was determined by adding the concentrations of sucrose, glucose, and fructose from HPLC analysis. However, because each component has different sweetness degrees, glucose, sucrose, and fructose values were weighted by factors of 0.75, 1, and 1.5, respectively [29]. Sourness was determined by adding each acid concentration after being divided by its own molar mass [30].

For astringency, a tannin/lignin test kit (HACH, CO, USA) was used to measure the total amount of phenolic compounds. Each tannic acid solution (1.25, 2.5, 5, 10, 15, 20, and 30 ppm prepared for standard curve) and 100-fold diluted wine samples were mixed with a drop of Folin-Ciocalteu reagent. Sodium carbonate solution (1 mL) was added and incubated for 25 min for color development. Transmittance at 410 nm was measured with the spectrophotometer (ColorMate, SCINCO, Seoul, Korea), and the values from wine samples were compared with the standard curve to measure the tannin values [31,32,33].

### 2.3. Colorimetric Sensor Array and Image Acquisition System

Eight types of non-specific chemical dyes were selected and conjugated on microbeads [12,13,14,15,16,17,18]. Those eight dyes were alizarin, calconcarboxylic acid, cresol red, crystal violet, fluorescein, methylthymol blue, phenol red, and xylenol orange. Amino-functionalized polystyrene microbeads with a 100–120-µm diameter were used as substrates to conjugate the selected dyes by using EDC (1-ethyl-3-(3-dimethylaminopropyl) carbodiimide) cross-linker [34,35]. Microbeads (100 mg) were washed twice in 10 mL of pH 7.4 PBS (phosphate buffered saline). After the second wash, beads were suspended in 10 mL PBS. Each dye (0.01 M) was suspended in 10 mL pH 7 MES (2-(*N*-morpholino)ethanesulfonic acid) buffer and 100 mg of EDC cross-linker were added and mixed at room temperature for 15 min. The dye-EDC conjugated suspension and microbead solution were combined and continuously mixed at room temperature for 2 h. Dye-conjugated beads were washed with deionized water [36]. All dyes, buffers, and a linker were purchased from Sigma-Aldrich (MO, USA). The sensor array template was fabricated with a silicon isolator (GRACE BIO-LABS, Bend, OR, USA) and attached to an adhesive-coated slide glass. Fifteen milligrams of each dye-bead conjugate was placed in each well separately. One hundred microliters of the wine sample were loaded into each well with a pipette.

**Figure 1:**
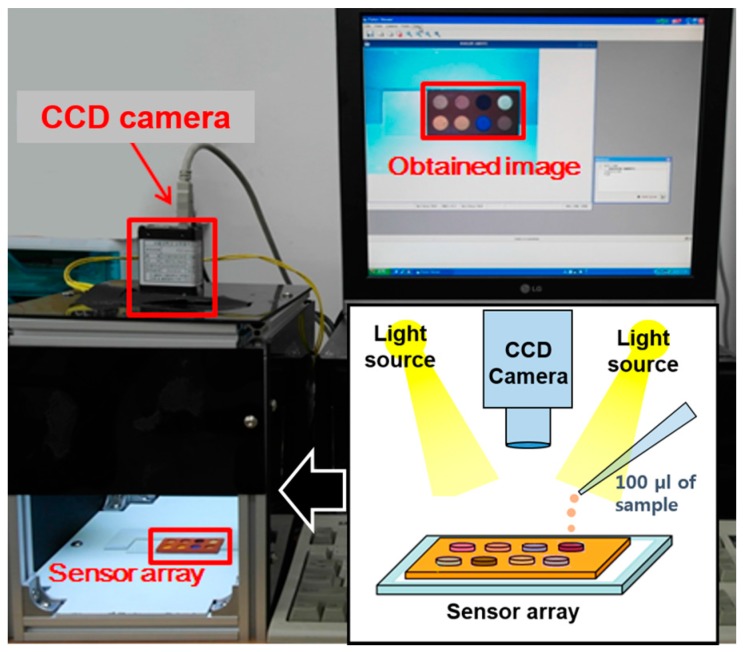
Image acquisition system for the colorimetric sensor array. Images of the taste sensor were taken before and after 100 µL of the white wine sample was placed on the taste sensor.

An image acquisition system was built with a charge-coupled device camera (CCD), personal computer, and light-emitting diodes (LEDs) (Figure 1). The CCD camera (A601fc, Basler, Ahrensburg, Germany) was mounted on top of the LED stands facing down and connected to the computer. A total of six LEDs (5050 SMD LED WPL, SSlight, Seoul, Korea) with 6 cd of luminance were installed in the stands to provide uniform lighting on the colorimetric sensor array while measurements were taken.

### 2.4. Data Acquisition and Analysis

Images of the sensor array were taken twice, before and 5 min after the addition of the wine samples. The RGB value change from each well was measured once and stored using Matlab code (2013b, MathWorks, Natick, MA, USA). This code recognized the location of the sensor chip and each well in the image to automatically gather 24 color data (eight wells × three colors). The color data from the first image (before loading the wine sample) was subtracted from that of second image (after loading the wine sample).

The color data obtained from the first sample set (n = 23) were analyzed with PCA (The Unscrambler v9.7, CAMO Software AS, Oslo, Norway) and validated in cross-validation mode.

The second sample set (45) was used for artificial neural network analysis to develop models to predict sample sweetness, sourness, and astringency from the color data. Open-source software for back-propagation of a neural network (Fast Artificial Neural Network Library, Ver. 2.2.0) [37] was used for programming. The ANN models for predicting each taste were created with combinations of configurations listed in Table 2. The performance of each developed model was evaluated by the coefficients of determination (*R^2^*) and RMSEP (root mean square error of prediction, Equation (1)).

(1) $$\text{RMSEP}=\;\sqrt{\frac{\sum_{i=1}^{N}{\left(k_{E}-k_{P}\right)}^{2}}{N}}$$

where *N* is the total number of data sets, *k_P_* represents the predicted taste value from the model, and *k_E_* is the taste value analyzed and calculated based on the experimental data.

**Table 2:** Combination of configuration variables for ANN (Artificial Neural Network).

Variable	Value
Algorithms	Incremental, Batch, RPROP, Quick PROP
Learning rate	0.5, 0.7, 0.9
Activation function	Sigmoid, Linear. Gaussian, Sin, Cos
Number of neurons in the 1st hidden layer	9, 11, 13
Number of neurons in the 2nd hidden layer	0, 9, 11, 13

## 3. Results and Discussion

### 3.1. Correlation of the RGB Color Change of Selected Dyes to Taste-Related Chemicals in the Wine

**Figure 2:**
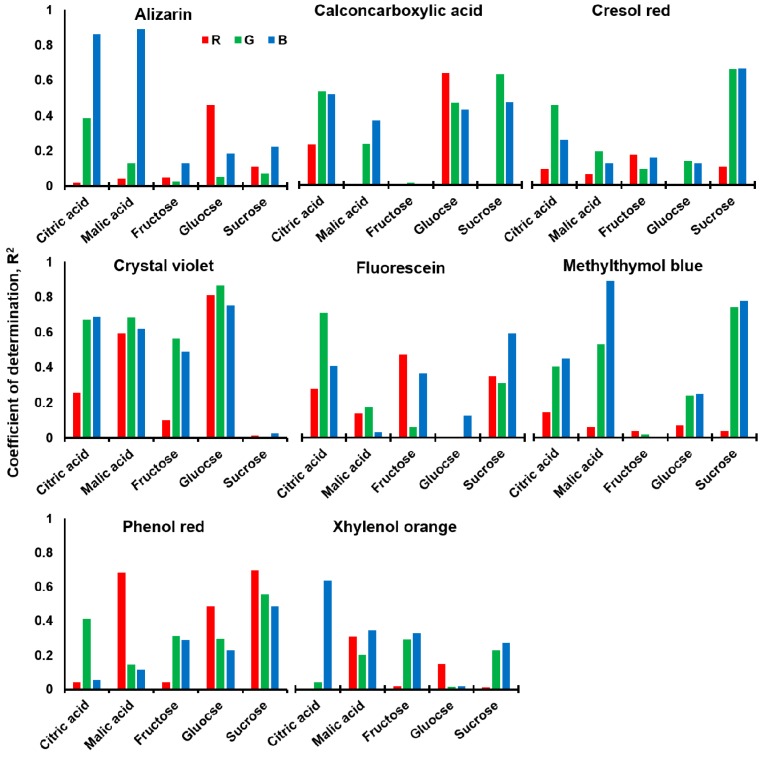
Coefficient of determination between the average absorbance of each dye-bead conjugate and the concentration of each taste-related chemical in terms of the red (620–780 nm), green (500–580 nm), and blue (450–500 nm) color values.

The correlation between the concentrations of the taste-related chemicals in the wine samples and the change in the RGB color value of dye-bead conjugates was evaluated. The absorbance spectrum (Colomate, SCINCO, Seoul, Korea) was measured from the mixture of each dye-bead conjugate and six different concentrations (citric acid and malic acid were from 0 to 13,500, and 8000 ppm; fructose, glucose, sucrose were from 0 to 5000 ppm) of each taste chemical (Supplement 2). Coefficients of determination (*R^2^*) of the average absorbance of red (620–780 nm), green (500–580 nm), and blue (450–500 nm) *versus* the concentration of taste-related chemicals were calculated (Figure 2). None of the dyes shared similar characteristics with each other.

### 3.2. Quantitative Analysis of Taste Components in White Wine Samples

Each white wine was tested by HPLC three times, and the values were averaged (Supplement 3). Twenty-three wines had sucrose levels of 32–4232 mg/L; glucose levels of 95–31,005 mg/L; and fructose levels of 376–64,258 mg/L. Malic acid levels were 311–46,291 mg/L; tartaric acid 996–21,191 mg/L; succinic acid 101–4588 mg/L; formic acid 96–1855 mg/L; lactic acid 0–1318 mg/L; citric acid 0–253 mg/L; and acetic acid 75–272 mg/L. According to the results, fructose is the major sugar component, while malic acid and tartaric acid were the primary organic acids. The amount of tannin analyzed by Folin-Ciocalteu reagent ranged from 72 mg/L to 573 mg/L.

**Figure 3:**
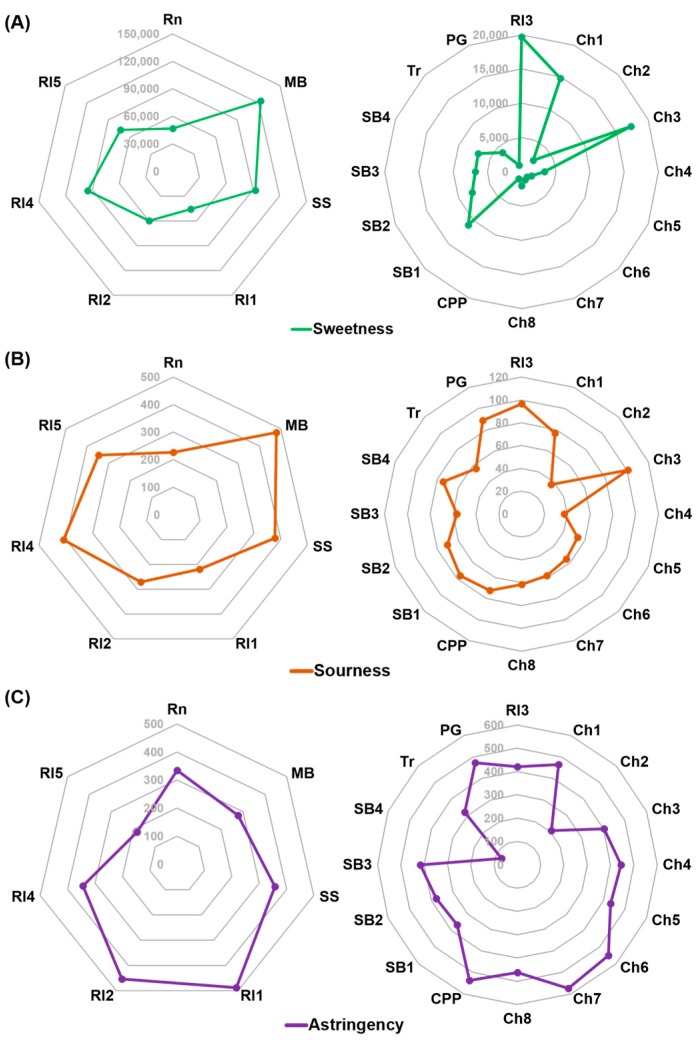
The sweetness (**A**); sourness (**B**); and astringency (**C**) of the white wine samples based on HPLC analysis and weighted. The left and right charts are for the sweet and less sweet wine samples, respectively.

The sweetness, sourness, and astringency of each wine were calculated using these analyzed values (Supplement 4). Adding the weighted amount of each sugar, the least sweet and the sweetest wines had sweetness values of 1029 and 123,019, respectively. Sourness was represented by the sum of the mole number of each organic acid, and the range of sourness values was 36–478. Astringency was assessed by the amount of tannin, and the range of astringency values was 72–573. Figure 3 shows the taste characteristics of individual wine samples based on HPLC analysis after weighting the values. Wine samples were simply divided into two groups by their sweetness and sourness. Seven wines were extremely sweet (sweetness: 46,800–123,019) and sour (sourness: 220–478) compared to the others (sweetness: 1029–19,705; sourness: 36–100) but with similar astringency.

### 3.3. Classification and Characterization of White Wine Taste from PCA

**Figure 4:**
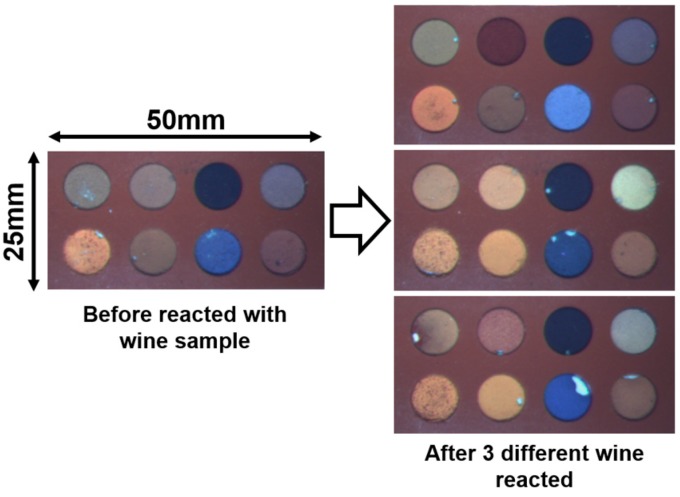
Images of eight dye-bead conjugates in the array before and after incubation with wine samples.

Images of the taste sensor before and after incubation (5 min) with three different white wines are shown in Figure 4. Matlab code captured the image, calculated the average RGB intensity from each of the eight wells before and after sample loading, and subtracted the final values from the initial. The 24-color (eight dyes × three color = 24) difference data from 23 wine samples were used for principal component analysis. Principal components (PCs) are variables generated in the course of performing PCA and have a linear relationship with the 24 color values. PC1 incorporates the most possible variance; PC2 has next most possible variance that is uncorrelated with the first component, and so on. The PCA output created a score plot of PC1 and PC2, which represents the relationship of each data point.

**Figure 5:**
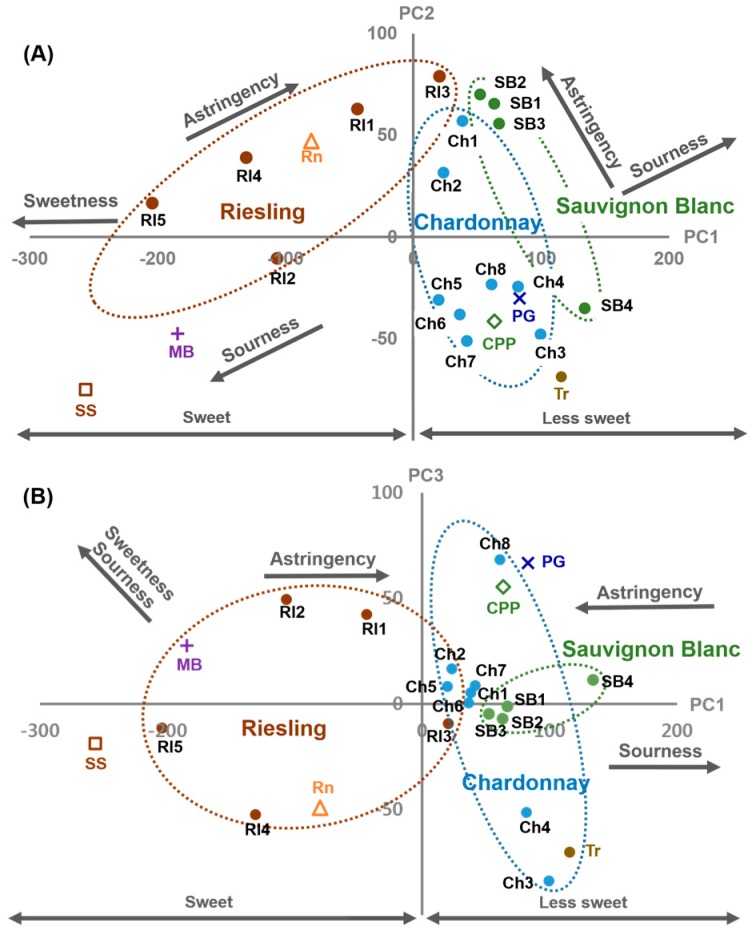
Score plot of PCA grouping wines with grape varieties and characterizing the taste on the plane of PC1 and PC2 (**A**); and PC1 and PC3 (**B**).

Color values from 23 white wines were plotted using PC1 and PC2 (Figure 5A) and PC1 and PC3 (Figure 5B) as axes. PC1 best described the total variables (55.96%), PC2 was uncorrelated with PC1 and represented 14.94% of the variables, and PC3 represented 11.66% of the variables. Principal component analysis reduced 24 color variables to three PCs explaining 82.56% of the total variables. As a result, Riesling, Chardonnay, and Sauvignon Blanc, which comprise a large number of the samples, formed a cluster. Rivaner (Rn) fell into the Riesling cluster because both have very similar taste characteristics; Rivaner is a hybrid of Riesling and Sylvaner [38]. The blend of 70% Chardonnay + 15% Pinot Grigio + 15% Pinot Blanc (CPP) was included in the Chardonnay group because the main grape variety of CPP is Chardonnay. Other white wines such as Moscato Bianco (MB), 80% Semillion + 20% Sauvignon Blanc (SS), Torrontes (Tr), and Pinot Grigio (PG) are distributed outside of the clusters.

### 3.4. Quantification of White Wine Taste Using ANN

We have developed three individual models to predict sweetness, sourness, and astringency from the color data using an artificial neural network. Color data from 45 wine samples were divided into training, validation, and testing groups. Thirty-five samples were randomly chosen for the calibration set, and the remainder of the samples were divided into a training set and validation set at a ratio of 7:3 to avoid over-fitting the model. Second, the remaining 10 samples were used for testing the developed model.

The ANN model was developed with combinations of five variables (four algorithms, three learning rates, five activation functions, three neurons in the first hidden layer, and four neurons in the second hidden layer). Each model predicted the taste value of 10 testing samples. These predicted values were compared to the actual taste value and used to generate the coefficient of determination (*R^2^*) and RMSEP. The model with the highest coefficient of determination and smallest RMSEP was selected as the best prediction model.

The following conditions produced the best predictor of sweetness: incremental algorithm, 0.9 learning rate, nine neurons, and sigmoid function on the first hidden layer; 11 neurons and sigmoid function on the second hidden layer; and sin function on the last layer. The following conditions produced the best predictor of sourness: QuickPROP algorithm, 0.9 learning rate, nine neurons, and sigmoid function on the first hidden layer; 11 neurons and Gaussian function on the second hidden layer; and sin function on the last layer. The following conditions produced the best predictor of astringency: batch algorithm, 0.7 learning rate, 13 neurons, and sigmoid function on the first hidden layer; 11 neurons and Gaussian function on the second hidden layer; and cos function on the last layer.

**Figure 6:**
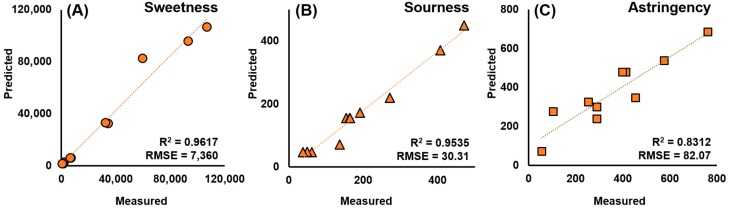
ANN model evaluation of measured and predicted sweetness (**A**); sourness (**B**); and astringency (**C**).

The performance of the best-performing models predicting the three tastes of white wines is shown in Figure 6. The coefficient of determination (*R^2^*) was 0.96, 0.95, and 0.83. The RMSEP was 7360, 30.31, and 82.07 for sweetness, sourness, and astringency, respectively. Considering the taste value range, the percentile of RMSEP on each taste is 6.03%, 6.86%, and 16.38%, respectively. Models developed from the ANN were able to predict the sweetness and sourness with minimal error. However, the astringency estimation model had worse performance compared to the results of the other two taste models. The inferiority of predicting astringency can be overcome by using precise measurements of tannins in wine samples.

## 4. Conclusions

In this study, we developed a colorimetric sensor array that can determine the sweetness, sourness, and astringency of white wine by principal component analysis (PCA) and artificial neural network (ANN). PCA reduced 24 color variables to three principal components and distinguished sweet and less sweet sets of white wine samples. Additionally, the trend or degree of sourness and astringency were plotted on a score plot. The PCA result can provide relative taste characteristics of other white wine samples. The ANN was used to develop the models that quantitatively predict sweetness, sourness, and astringency. Sweetness and sourness were predicted very accurately (*R^2^* > 0.95), and astringency was predicted well enough to provide an approximate level. The developed colorimetric sensor array for white wine has high potential for identifying the taste components of white wine and can contribute to the wine industry by providing the level of sweetness, sourness, and astringency to the consumer, enabling choice of wine based on taste preferences.

In conclusion, the taste sensor developed in this study can provide cost-effective analytical methodology to quantify the taste of liquid food as well as to discriminate the species in the food industry.

## Supplementary Files

Supplementary File 1
